# A Retrospective Proof-of-Concept Study of the Impact of Point of Care Ultrasound During Short-Term Surgical Missions

**DOI:** 10.24908/pocusj.v10i02.18453

**Published:** 2025-11-17

**Authors:** Harsh Sule, Rolando Valenzuela, Vennila Padmanaban, Enoch Obeng, Georgia Davies, Francisco Alvarado, Ziad Sifri

**Affiliations:** 1 Department of Emergency Medicine, Rutgers New Jersey Medical School, Newark, NJ, USA; 2 Department of Emergency Medicine, Renaissance School of Medicine, Stony Brook University, Stony Brook, NY, USA; 3 Department of Surgery, Rutgers New Jersey Medical School, Newark, NJ, USA

**Keywords:** Global surgery, Short term surgical mission, Point of Care Ultrasound, Sierra Leone, Ghana, POCUS

## Abstract

Point of care ultrasound (POCUS)-use during short-term surgical missions (STSMs) to resource-limited settings has not been well studied. We conducted a retrospective analysis of POCUS use during the perioperative course of patients undergoing definitive surgical treatment over the course of two STSMs. A total of 58 perioperative POCUS exams were performed by emergency physicians on our team. Operative findings correlated with POCUS results in 90% of cases that underwent surgery, while surgery was deferred based on POCUS findings in 33% of scans. Our findings suggest that POCUS is a portable, rapid and cost-effective modality that can be used in a focused manner in the perioperative period. Specifically, our inter-disciplinary experience and results demonstrate that POCUS-use has a positive impact on patient safety and quality, and optimizes the use of valuable resources and time.

## Introduction

Over the past several decades, great strides have been made in global health. While the need for globally significant work is critical, the impact has often been on individual diseases. Treatment for surgical conditions, including a broad range of diseases that represent approximately 30% of the global burden of disease and span 100% of disease sub-categories, remains out of reach for the majority of the world's population [1]. Timely access to surgical care with local human capacity to provide surgical care is essential. However, the reality remains that there is a large gap between current needs for surgical care and the ability to train and deploy local personnel in a rapid manner. This gap is often filled by short-term surgical missions (STSMs). These are most effective when undertaken in an ethical manner with awareness of local needs, adequate pre- and post-operative evaluation and care, high-quality delivery of care, and concurrent educational initiatives. STSMs can also make a tangible positive impact on the patient population including on disability-adjusted life years (DALYs) [2,3].

Imaging is amongst the many challenges of STSMs. Venues that host STSMs often have few, if any, imaging options. While plain film radiology (x-rays) may be available, their utility is limited. Computed tomography scanning (CT scans) may be available at some advanced centers in urban areas, but have limitations related to location, cost, and quality, and may rarely be accessible in rural districts. Ultrasound (US) is an effective modality that “provides the capacity for a broad range of rapid diagnoses” [4]. US is also non-invasive, cost-effective, and safe for patients and technicians when compared to other imaging modalities [5].

US in resource-limited settings has underpinned the triage of true surgical emergencies. Practitioners have identified abdominal aortic aneurysms and hepatobiliary conditions such as hepatic abscess and cholecystitis [5,6]. When evaluating groin pathology, US can be useful in adjudicating the presence and type of groin hernia in the setting of equivocal clinical hernia findings, and can help diagnose postoperative complications such as hematoma and seroma [7]. US has also been noted to change the management of patients with regard to performing surgical procedures like caesarean sections, biopsies, and even minor surgeries after certain findings were effectively visualized [6,8]. Modern surgical diagnoses are based on thorough history and physical exams that are supported by imaging findings. US has special utility in general surgical, gynecologic and urologic conditions [9]. This is even more starkly delineated in Global Surgery. US exams performed by clinicians and interpreted immediately at the bedside are considered point of care ultrasound (POCUS).

The 1990s saw the advent of “portable” US: US that was the size of a backpack and an ideal size for hand-carriage to low and middle-income countries (LMICs) [10]. This has now further improved with the introduction of Ultrasound-on-Chip™ technology that further optimizes portability and flexibility of use [11]. In LMICs, the World Health Organization described how the demand for adjunctive imaging modalities can be resolved using diagnostic US [9]. In a low-resource country like Ghana, POCUS was noted to be useful not only because of its portability and availability, but its ability to immediately detect critical findings [12].

POCUS-use has a unique niche in global surgery. Focused Assessment with Sonography in Trauma (FAST) exams can screen for intra-abdominal hemorrhage in the pre-hospital setting and in emergency departments globally, and cardiac POCUS is helpful in the pre-operative evaluation of patients as well as in those with cardiac compromise [8]. The use of POCUS in Uganda for breast imaging has helped to decrease how frequently biopsies are performed for normal or benign findings [13]. Although there is growing literature that hand-carried USs used for POCUS examinations have diverse practical implications for surgical diagnosis, they have not been well studied for use during STSMs to resource-limited LMICs [5].

The STSM model aspires to bring safe surgical care to LMICs that have a lack of health infrastructure and often lack the same standard of care as the United States for the quality of diagnostic imaging [2,14]. Missions are typically 1-2 weeks in length, carried out by a multidisciplinary team of healthcare professionals in coordination with local providers. This has required adapting technologies such a POCUS for novel applications, given that diagnostic modalities are limited [15–17]. This study examines the utilization of POCUS in pre-operative, intra-operative and post-operative periods.

## Materials & Methods

We conducted a retrospective analysis of the use of POCUS during the pre-operative, intra-operative and post-operative course of patients undergoing definitive surgical treatment over the course of STSMs to Kabala, Sierra Leone in December 2017 and Mampong, Ghana in September 2018 and 2019. The missions were conducted by volunteers with the International Surgical Health Initiative (ISHI), a non-governmental organization that conducts annual trips to the hospitals and has longitudinal relationships with local personnel in several countries. Each trip was conducted over a two-week period with arrangements for post-operative care.

Patients were recruited and pre-screened for possible surgical intervention by local hospital staff at Kabala General Hospital (KGH) in Sierra Leone and at Tetteh Quarshie Memorial Hospital (TQMH) in Ghana. The range of possible elective surgeries included, but were not limited to, herniorrhaphy, hydrocelectomy, hysterectomy, removal of lipomas and other benign soft tissue masses. These hospitals function in resource-limited conditions, at times with one physician and some community health officers. Diagnostic tools are limited to physical exam skills and a single x-ray machine unless patients pay to obtain their own advanced imaging at local private outpatient centers—something most patients cared for within the STSMs are unable to do.

For the purposes of the 2017 and 2018 missions, two portable US machines were obtained as short-term donations from Fujifilm (Bothell, WA, USA), including the M-Turbo model and the portable IViz model (3 probes were utilized for these studies: Curvilinear (2-5 MHz), Linear (6-13 MHz) and Phased Array (1-5 MHz). The September 2019 mission to Ghana utilized Butterfly iQ® (Burlington, MA, USA) probes (3 software settings to mimic the above 3 probes) with Apple (Cupertino, CA, USA) iPads® that were provided as short-term donations by the Rutgers New Jersey Medical School's Department of Emergency Medicine. These machines were used for diagnostic purposes by emergency medicine faculty members and/or by emergency medicine residents with formal POCUS training per their residency curriculum with live/hands-on supervision by emergency medicine faculty. The emergency medicine faculty members were all POCUS-certified based on their own residency training but were not specifically fellowship-trained in clinical US.

**Figure 1.**
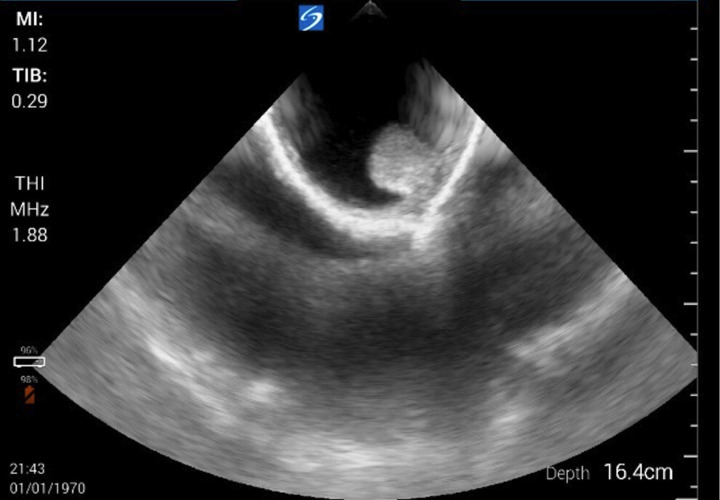
Pre-operative point of care ultrasound (POCUS) examination of unilateral scrotal swelling revealing hydrocele.

**Figure 2.**
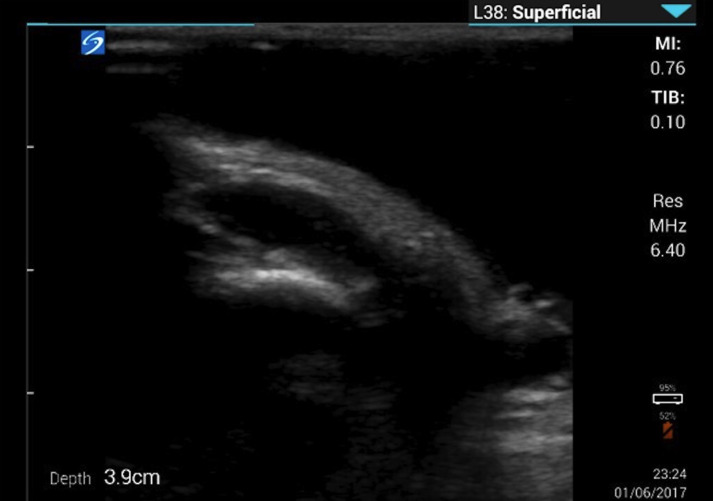
Pre-operative point of care ultrasound (POCUS) examination of unilateral scrotal swelling revealing both hydrocele and inguinal hernia components.

**Figure 3.**
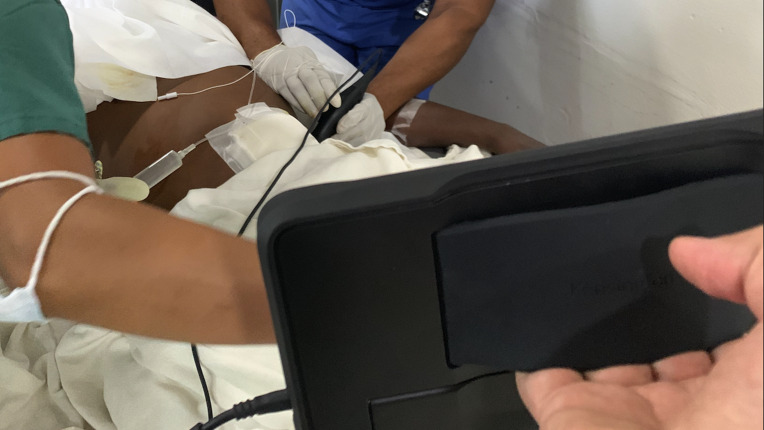
Post-operative use of point of care ultrasound (POCUS) for performance of Transversus Abdominis Plane (TAP) block.

**Figure 4.**
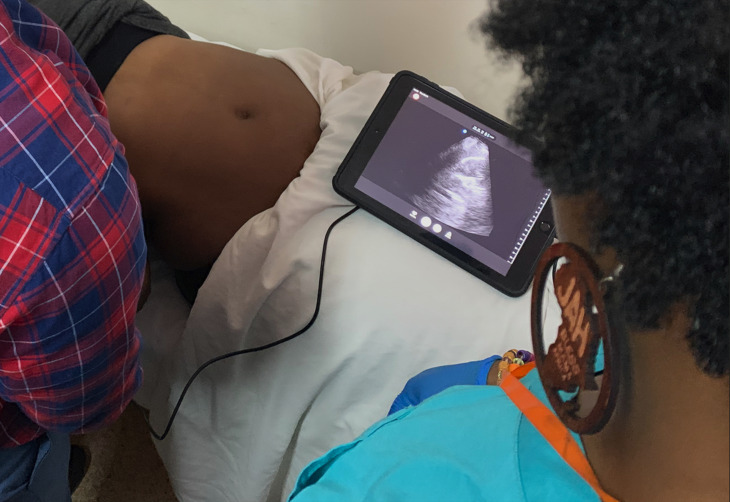
Use of point of care ultrasound (POCUS) for evaluation of a patient with abdominal pain.

POCUS served two purposes: First, to definitively diagnose pathology that could benefit from surgical intervention, and second, to enhance operative planning and reduce risk while also optimizing the use of scarce supplies/resources. The emergency physicians provided support to the surgical teams on day 1 of the mission during patient triage, as well as intra- and post-operatively when requested by the surgical teams. Separately, POCUS was used to assist local personnel in other medical decision making including emergency and obstetrical care, but details of those uses are not reported in this study. (Figures 1-4)

The number and types of POCUS examinations performed were recorded, as well as the preliminary diagnosis resulting in the request for a study, the post-POCUS diagnosis and finally the post-operative diagnosis. Instances when there was a change in or impact on the operative plan were noted as well.

## Results

Over the course of three ISHI missions, a total of 58 POCUS examinations were performed as part of the perioperative clinical evaluation related to questions regarding the exact pathology and/or concerns regarding operative risk (Table 1).

**Table 1.** Pocus and Surgical Summary Data

POCUS Views	Sierra Leone 2017	Ghana 2018	Ghana 2019	Total
**# of patients evaluated/tiraged for surgery**	98	124	108	330
**# of operations performed**	65	69	80	214
**# of POCUS studies performed**	15	26	17	58

Studies that were performed assessed for:

- Inguinal/scrotal pathology: differentiating between hernias, hydroceles and mass lesions.
- Abdominal wall hernia: size of a defect to determine appropriateness for intervention on the short-term surgical mission (STSM) and optimize use of supplies such as mesh.
- Uterine fibroid: identify size, location and distance from the cervix to determine appropriateness for intervention on the short-term surgical mission (STSM).
- Soft tissue mass: assess size, depth, adjacent tissue involvement and differentiate pathology such as lipoma, sebaceous cyst, etc.
- Prostate: assess size to determine appropriateness for intervention on the short-term surgical mission (STSM).
- Cardiac function: guide in determination of operative risk.
- Other: assess operative need and risk due to concurrent pathology, as well as investigate intra- and post-operative complications such as hematomas and blood flow.

**Figure 5.**
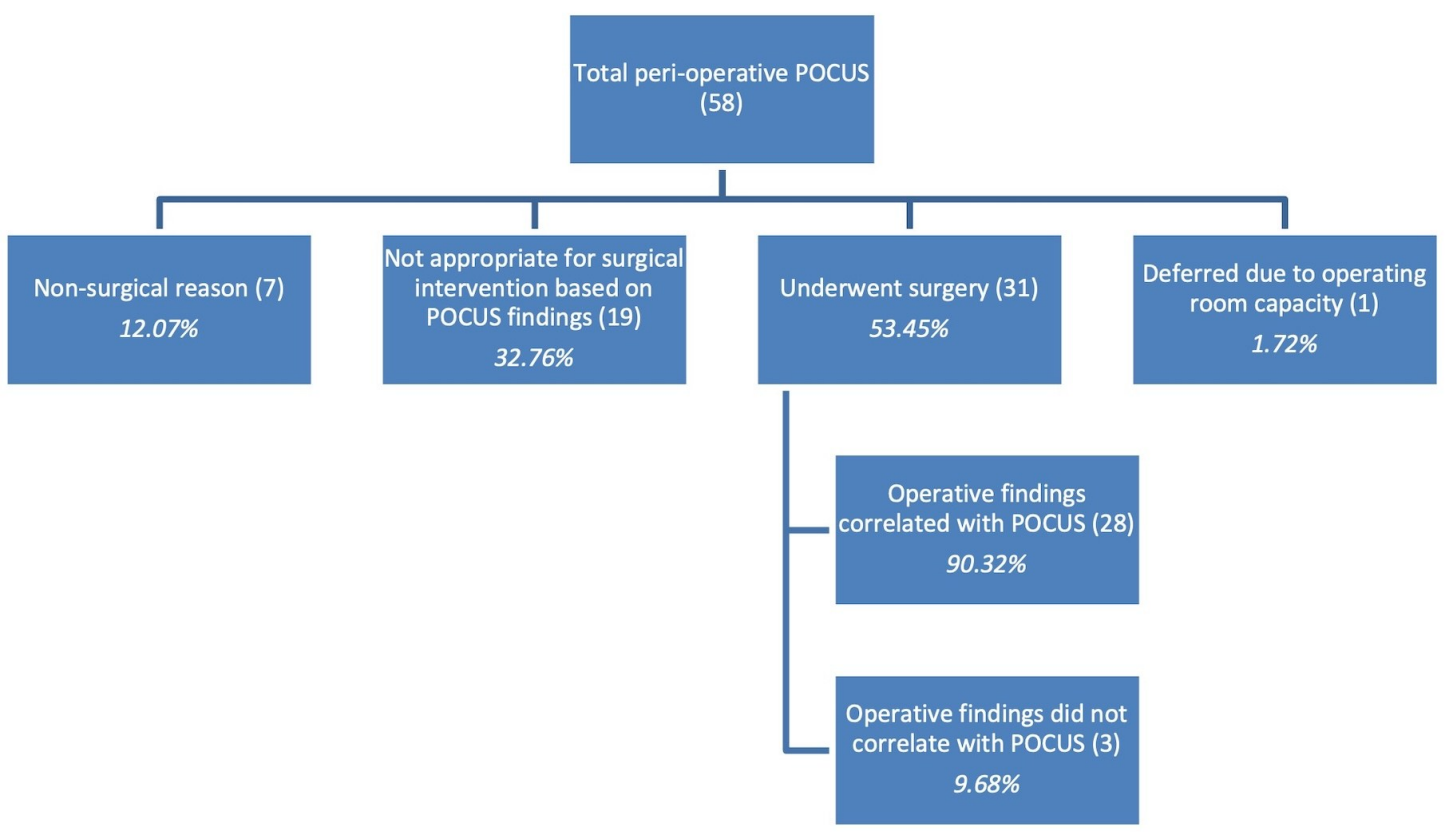
Outcomes of peri-operative POCUS

Figure 5 outlines the results of the perioperative POCUS studies performed on the three missions. Of the 58 POCUS examinations performed, 7 (12.07%) were for non-surgical reasons but were still related to their pre-operative evaluations. Specifically, these included cardiac evaluation and assessment of other pathology that may influence surgical risk. Nineteen studies (32.76%) resulted in findings that deemed operative intervention during the STSM to be inappropriate. This was either due to the scale of resources and equipment necessary, anticipated surgical time or identification of pathology that was not amenable to intervention on the STSM. Eleven of these cases demonstrated pathology that differed from the initial clinical examination, but this was not confirmed operatively. One case (1.72%) of benign prostatic hyperplasia was appropriate for surgical intervention but was cancelled due to scheduling challenges and operating room capacity. Of the 31 cases (53.45%) that underwent surgery, 28 cases (90.32%) demonstrated findings that correlated with the POCUS examination. In three cases (9.68%), the operative findings did not correlate fully with the POCUS findings:

- Hernia defect size: a hernia was measured by POCUS at 3 cm but was found to be 5 cm.
- Testicular mass: a hydrocele appropriately identified by POCUS was found intraoperatively, but an additional small mass that was not found on POCUS was noted during the operation.
- Hydrocele: a large hernia was interpreted by POCUS but intraoperatively it was found to be a large hydrocele. The hydrocele was noted to have thick and irregular walls that may have led to the misinterpretation.

In addition to the above studies, POCUS was used in the post-operative period to evaluate potential complications such as the etiology of fluid collections, confirm the absence of active blood flow in an incisional hematoma, assess urinary bladder volume in cases of urinary retention, and perform nerve blocks for pain management.

Finally, over 50 POCUS examinations were performed for a wide range of clinical uses in the emergency department, ambulatory clinics, and inpatient and maternity wards in support of local healthcare personnel.

## Discussion

The use of POCUS in LMICs, where lack of infrastructure is a barrier to diagnostic medicine, is not well-described in the global health, global surgery, or emergency medicine literature. Its use has been discussed in obstetric screening, however, it has not been described for screening and assessment of non-emergent surgical pathology [16]. While hand-carried US equipment is contingent on battery power and is subject to overheating in tropical climates, it is still advantageous for its portability, low resource utilization, and relative cost-effectiveness. POCUS provides real-time feedback and obviates the need to transport the patient to obtain imaging [9].

Our experience and results demonstrate that the use of POCUS in conjunction with multi-disciplinary teams in LMICs for STSMs has a synergistic effect with a positive impact on patient safety and quality. Our data demonstrates that POCUS can increase the accuracy of initial diagnostic impressions and thereby reduce unnecessary surgical interventions. Furthermore, the use of POCUS can optimize the distribution of valuable resources and time. Specifically, our data demonstrated an accuracy of 90% based on surgical correlation of POCUS findings and that 33% of plans for surgery were changed based on POCUS findings during the triage process. Our work demonstrates that providers such as emergency physicians trained in a focused manner can effectively identify specific pathology with a low rate of error and can thereby function as effective partners in STSMs. POCUS therefore has a positive impact on the diagnostic capabilities of the team and allows for improved patient outcomes and resource utilization.

## Study Limitations

The study was limited primarily by the retrospective nature of the data collection. Future iterations should use a prospective study tool to obtain more robust data. Additionally, the POCUS examinations in Sierra Leone were performed by a single physician with no secondary readings, while those in Ghana involved multiple physicians viewing the same study and arriving at a consensus. Due to technical limitations, we were unable to save the images for later review. This would have been helpful in cases where the operative findings did not fully correlate with the POCUS interpretation so that we could identify whether there were errors in technique or interpretation.

## Conclusions

From the range of diagnostic uses for POCUS in just a few STSMs in Sub-Saharan Africa, a model emerges for its inclusion as standard practice on surgical missions. We plan to further study the impact of POCUS on patient outcomes in surgical disease. In addition to its broad practical utility in the areas of surgical and emergency disease screening, POCUS can be the focus of clinical collaboration amongst global health specialties. Moreover, it may have utility in bedside teaching and capacity-building, which are the educational tenets of STSMs that have everlasting impact [17].
